# Development and testing of an explorative BPM acceptance model: Insights from the COVID-19 pandemic

**DOI:** 10.1371/journal.pone.0259226

**Published:** 2021-11-04

**Authors:** Tahir Ahmad, Amy Van Looy

**Affiliations:** Department of Business Informatics and Operations Management, Faculty of Economics and Business Administration, Ghent University, Ghent, Belgium; University of Salento, ITALY

## Abstract

When emerging technologies transform an organization’s way of working, explorative business process management (BPM) becomes a new challenge. Although digital innovations can boost process efficacy and business productivity, employees do not necessarily accept the implied work changes. We therefore looked at the increased digitalization efforts during the COVID-19 lockdowns, during which employees were forced to drastically rethink work by heavily depending on technology for communication and almost all business tasks. This global setting allowed us to scrutinize disruptive work changes and how employees can cope with disruptive work adaptations. We also looked into the explorative skillset needed to adapt to these changes. To theorize about an explorative BPM acceptance model, eleven hypotheses were supported based on a solid theoretical foundation. We followed a quantitative research design using partial least squares for structural equation modeling (PLS-SEM) at the university administration settings in two regions, including purposive sampling. Data analysis covered both a measurement model assessment and structural model assessment. Our findings reveal that employees’ perceived work modalities, feeling creative and feeling flexible are more promising features than perceived influence and attitude related to explorative work and skill development. We also offer novel insights into explorative business process management (BPM) skills, and which skills are more productive in uncertain or dynamic working conditions. This research is a learning path for managers struggling with flexible or competitive business environments, and more specifically to facilitate employee willingness.

## 1 Introduction

Working with emerging technologies in Industry 4.0 requires a more agile mindset of employees to become able to explore innovative opportunities [1]. Changes in technology and processes are strongly interlinked since new technologies have the potential to disruptively transform a business process [2]. However, when work patterns or routines are being changed to obtain a competitive advantage or to overcome an economic crisis, it remains difficult to promptly overcome the impediments related to those new work mechanisms [3]. Especially employees tend to resist against work adaptabilities because of distrust [4], which can even result in decreased employees’ confidence in the existing pattern of work routines and a lack of flexibility for the new ones. Therefore, change management is crucial under circumstances when employees are forced or need to adopt new ways of doing business. In response, theories have emerged to support users’ technology acceptance, such as the original and well-established Technology Acceptance Model (TAM) [5].

Although the business process management (BPM) discipline fosters an innovative approach (i.e., by means of reengineering) [6], it typically focuses on incremental work changes such as total quality management (TQM), Lean manufacturing, and Six Sigma [7]. Alternatively, the literature presents ambidextrous BPM as a mix of traditional (or exploitative) BPM and explorative BPM characteristics [6], in line with the ambidexterity theory of [8]. The concept of ambidextrous BPM was initially explained by [9] when predicting the future of BPM. Similarly [10], positioned ambidextrous BPM on the organizational level while linking it with big data management, whereas [11] studied both BPM exploitation and exploration in the light of IT capabilities. To our knowledge [12], were the first to explain the research potentials of explorative BPM. Meanwhile, BPM ambidexterity is experienced when offering work alternatives for continued usage after the COVID-19 pandemic [13].

This study aims at developing and testing a theory for explorative BPM acceptance. More specifically, based on a survey in university administration settings, we uncover employees’ perceptions about new ways of working by combining the theories of IT acceptance (i.e., TAM) [5] and ambidexterity theory [8]. We specifically re-used TAM’s main constructs (i.e., PU or perceived usefulness, and PEOU or perceived ease-of-use) and translated them into employees’ (1) perceived usefulness of explorative work changes and (2) perceived ease-of-use of explorative work changes. By extending the idea of TAM into an explorative BPM acceptance model (EBAM), we investigate employees’ work features along with their explorative BPM skills in order to evaluate the extent to which those adapted work practices are perceived as better than those in the pre-crisis of COVID-19, and thus by predicting their continuation in the post-crisis period. For this purpose, we classified actual explorative work features into (a) perceived work modalities, (b) perceived work distractions, and (c) perceived work implications. Based on the intended explorative BPM acceptance model, we target the likeliness or attitude for using explorative BPM skills, given the fact that such explorative skills have been mandatorily applied due to the sudden and mandatory COVID-19 lockdowns worldwide. Actual explorative BPM skills are classified as follows: (1) feeling creative (2) feeling opportunistic, (3) feeling flexible and (4) feeling adaptive to change. Both work features and skills are considered as exogenous variables in our explorative BPM acceptance model. Moreover, we incorporated perceived top management support as a third exogenous construct for having evidence from technology adoption studies as well [14].

So far, only limited research has been done on the explorative side of BPM ambidexterity, particularly on BPM skills and their practical adoption of explorative changes. To tackle this gap, our study focuses on the global lockdown periods that were caused by the COVID-19 pandemic during 2020 and 2021, and which suddenly transformed the work modalities in almost all organizations worldwide. Since the COVID-19 pandemic enforced practitioners to drastically redesign their business processes in a fast manner, the lockdown crisis has raised the opportunity to draw new insights into explorative BPM efforts while being less affected by employee resistance. Our purpose is to investigate employees’ attitude (i.e., in the sense of their willingness) to continue with (1) the explorative work adaptations and (2) explorative skill development in the years after the lockdowns (i.e., near future). The related research question is:

How can employees better adopt uncertain work changes by improving their explorative BPM skills?

By combining the two theoretical perspectives of TAM and ambidexterity theory, we contribute to BPM ambidexterity by focusing on its explorative part, which is still under-investigated. Managers and BPM practitioners (i.e., IT consultants and business analysts) are offered lessons learned about process innovation and explorative BPM during the COVID-19 lockdowns in respect of their human capital. Research novelty includes the development of an explorative framework for highlighting the factors and skills that can disrupt employees’ attitude and work behavior. The article proceeds with the theoretical background in Section 2. Then, the theoretical model is developed (Section 3), followed by the research method to test the model (Section 4). Subsequently, the results are presented (Section 5) and discussed (Section 6). We end with concluding remarks in Section 7.

## 2 Related literature

We first describe the work adaptations caused by the COVID-19 lockdowns, and underpin them by two related theoretical perspectives, namely ambidexterity theory and IT acceptance theories. These two theoretical angles will be applied to the work adaptations during the COVID-19 lockdowns and the related mandatory teleworking. Hence, the business climate during our study was characterized by a severe pandemic disease, which was caused by a virus that emerged in China at the end of December 2019 [15]. Governments worldwide unexpectedly had to impose preventive measures such as social distancing, self-isolation and working from home [15]. These lockdowns introduced new work change modalities in a sudden and drastic manner, since employees were forced to telework and communicate via videoconference technologies. In this situation, both traditional (exploitative) and explorative work skills turned out to be essential, with the management of tasks or processes being categorized as ambidextrous BPM.

Ambidextrous BPM focuses on a combination of explorative methods and techniques for managing a business process along with more incremental or exploitative methods and techniques [9]. When emerging technologies cause disruptive changes, organizations simultaneously adopt novel ways of doing business in parallel with their existing information systems [16]. From the perspective of digital innovation, an explorative BPM approach refers to being more adaptive, flexible, informal, open, agile, and creative for uncertain business processes to gain a relative competitive advantage [12]. For example, organizations such as Airbnb, Netflix and Uber operate with more creative and flexible business processes than their traditional variants (i.e., hotels, TV and taxi companies). However, during the COVID-19 lockdowns, also many traditional organizations attempted to reshape their business processes by exploring more process deviations from routines [17, 18]. For instance, many organizations started to invest in webshops and chatbots [19].

While explorative BPM is characterized by multiple aspects such as design thinking and new business models [9], our study primarily focuses on the required explorative BPM skills. A purposive literature review was conducted, during which 14 recent research articles were reviewed for common characteristics of explorative BPM skills.

Given the burden of employee resistance, technology acceptance and adoption studies have been predominately observed in prior management information systems (MIS) research. More specifically, a dedicated technology acceptance model (TAM) [20] was derived from the general theory of reasoned action (TRA) to incorporate the users’ acceptance of a new technology [21]. TAM includes perceived usefulness (PU), perceived ease-of-use (PEOU), intention to use and usage behavior. PU refers to the use of a technology or system to improve job performance, whereas PEOU is about the use of such IT with less efforts [22–24]. TAM describes the impact of PU and PEOU on “usage behavior” and “intention to use”, and boosts these concepts as mediating the relationship. According to TAM, PEOU is also directly affecting PU [20, 25]. Afterwards, TAM2 has added some extensions to the original TAM constructs but mainly by adding external variables, namely: (1) subjective norms, (2) image, (3) job relevance, (4) output quality, (5) result demonstrability, and (6) the moderating role of “experience” and “voluntariness” [25]. [21] have given a Unified Theory of Acceptance and Use of Technology (UTAUT), which was derived from eight different theories of user behavior in change dynamics. It contains four central elements of technology acceptance, such as performance expectancy, effort expectancy, social influence, and facilitating conditions [26].

Similarly, top management support has been previously described as a positive trigger or success factor for a technology adoption [27] and for accepting change or innovation [28]. But when managerial control becomes weaker (e.g., in telework, especially for team work), employees’ perceptions about top management support is more likely to change [29]. Top management support or involvement is considered a success factor for technology adoption [30]. For instance [30], operationalized top management support in combination with TAM for the adoption of ITIL. Another example is the acceptance of electronic health records by incorporating UTAUT characteristics with top management support [31].

Following TAM-related studies, many researcher also investigate attitudes while translating TAM into various acceptance scenarios as an alternative to “behavioral intention to use”. For instance, [32] used “attitude towards use” in an earlier post-COVID study about internet banking usage by using PU and PEOU as predictive constructs. Similarly [33], applied the TAM constructs to investigate users’ attitudes towards using an e-learning system. Therefore, employees’ attitude towards future explorative work (i.e., after the COVID-19 lockdowns) can also be used.

Finally [34], discussed the users’ attitude towards technology skills during a web-based project and highlighted that a single project can already stimulate skill development through an adopting attitude. Also background and expertise in a relevant business field are likely to improve this adaptive attitude for a specific skill set [35]. Additionally, a positive attitude in a work group can result in greater perceived benefits towards skill development [36]. Consequently, employees’ attitude towards future explorative BPM skill development can be considered as well.

## 3 Hypothesis development

**Fig 1:**
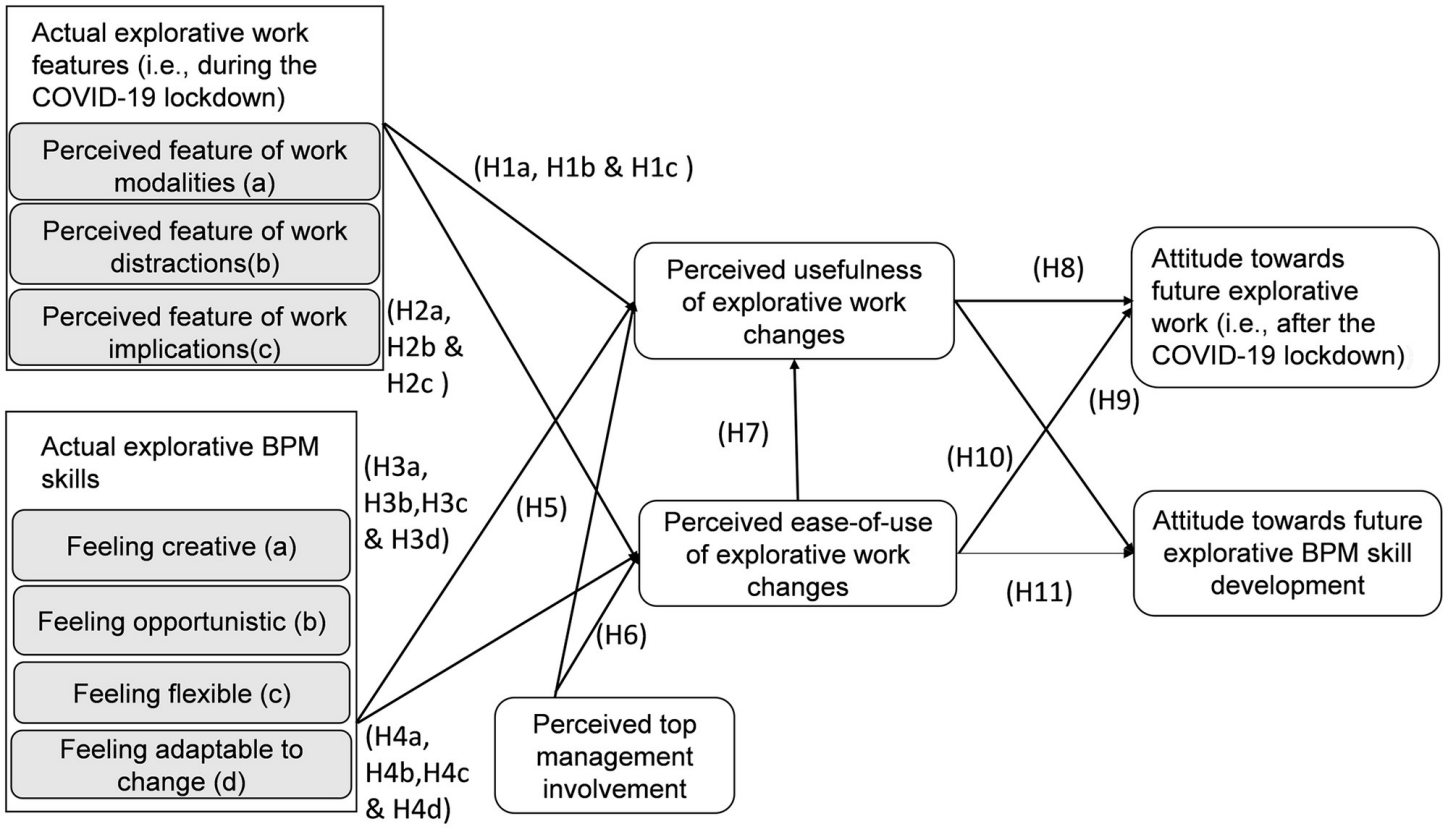
Explorative BPM acceptance model (including the COVID-19 lockdowns perspective).

Based on the theoretical underpinnings of ambidexterity theory and TAM (Section 2), we have developed an initial research model that represents an explorative BPM acceptance model (Fig 1).

### 3.1 Explorative work features

The literature concretizes the related work features for this exploration of work changes (i.e., modalities, distractions and implications) as follows.

During the COVID-19 lockdowns, many employees were obliged to adapt new ways of working remotely from home by using telephone, laptops, tablets and other facilitating tools [37]. Moreover, working hours and workable processes became much more flexible than regular routines, which urged employees to deviate from their normal work structures. For instance, while many people turned temporarily unemployed (i.e., either fulltime or part-time), some sectors rapidly grew such as pharma and face sheet manufactures (IBISWorld, 2020). In sum, the COVID-19 crisis forced all employees to deviate from existing processes and to explore new paths that favor teleworking and less bureaucracy.

Work adaptations during COVID-19 can also lead towards distractions from work routines. For instance, teleworking is flexible but can be challengeable for employees who are not acquainted to it [38]. More specifically, while online working from home weakens control over employees and increases work flexibility, some challenges during the COVID-19 lockdowns were related to internet-based technology (ICT) failures and a lack of adaptation for employees who were less experienced with online communication tools (e.g., ZOOM) or digital productivity platforms (e.g., Microsoft Teams) [39]. Even though working from home can enhance a work-life balance because employees can spend more time with family [40], distractions can also be caused by children or housemates [41]. Alternatively, setting priorities between work and home can cause home-to-work conflicts, e.g., going to the grocery or attending an online discussion. Finally, social isolation from co-workers can decrease the moral of team work for employees who perform better in teams, namely with the motivation and support from peers [42]. Since such challenges can reduce efficiency, a balance between flexibility and efficiency remains important.

Another feature that characterized work during COVID-19 was related to individual efficiency in work modalities, which we considered under the label of work implications. We refer to work change implications as efficiency in work patterns. For instance, autonomous employees may feel more comfortable without hierarchical monitoring or control, and therefore perform better with self-monitoring [43]. Alternatively, some employees perform better in online meetings and work process as compared to a physical office because online meetings can be more focused and effective [44].

To examine the impact of actual explorative work features (Section 2.1) on “perceived usefulness of explorative work changes”, we developed one hypothesis including three features, namely:

H1: The perceived features of work change modalities (H1a), distractions (H1b) and implications (H1c) have a positive impact on one’s perceived usefulness of explorative work changes.

Similarly, to investigate the impact of actual explorative work features (Section 2.1) on “perceived ease-of-use of explorative work changes”, we developed this hypothesis:

H2: The perceived features of work change modalities (H2a), distractions (H2b) and implications (H2c) have a positive impact on one’s perceived ease-of-use of explorative work changes.

### 3.2 Explorative BPM skills

From the literature, we derived four groups of skills for employees, namely: (1) feeling creative, (2) feeling opportunistic (3) feeling flexible and (4) feeling adaptive to change (see Table 1).

**Table 1:** List of articles for explorative BPM skills evidence.

Sr No.	Author(s) & Year	Study focused on…	BPM skill discussed			
			Feeling Creative	Feeling Opportunistic	Feeling Flexible	Feeling adaptive to change
1.	(Giudice et al., 2018)	Ambidexterity performance based on IT, production and logistics			×	×
2.		Directions for future development of BPM, specifically for explorative BPM	×	×		
3.	(Kohlborn et al., 2014)	Original concept of explorative BPM and comparison between exploitative and explorative BPM characteristics		×	×	×
4.	(Rosemann, 2014)	Systematic understanding of explorative BPM Triple diamond model containing business, technology and their integration	×	×	×	×
5.	(Grisold, Gross, Röglinger, Stelzl and vom Brocke, 2019)	Studied the effect of big data on ambidextrous organizations’ agility. Highlighted significance of BDA-capable BPMS.		×	×	×
6.	(Rialti, Marzi, Silic and Ciappei, 2018)	Comprehensive book on BPM		×		×
7.		Business and IT alignment was discussed under TOE framework. BPM exploration is also a key point.				×
8.	(Dumas et al., 2013)	Creating a balance and flexible business environment through IT and ambidexterity			×	×
9.	(Gabryelczyk, 2018)	Testing explorative and exploitative BPM with IT capabilities	×	×	×	×
10.	(Heckmann, Hsu, & Mädche, 2016)	Impact of Big data on BPM and Pharma industry data management		×		
11.	(Ferraris et al., 2018)	Utilization of resources of IT for flexibility and balance between explorative and exploitative BPM		×	×	×
12.	(Festa et al., 2018)	Difference between explorative and exploitative BPM			×	×
13.	(Heckmann & Maedche, 2018)	Integration between explorative and exploitative BPM enhance performance	×	×	×	×
14.	(Jilani, 2020)	Business process management adoption in family businesses and SMEs		×	×	×

The first explorative BPM skill under study is “*feeling creative”* at the workplace. Research has shown that professionals with an explorative mindset tend to be more creative and able to work without heavy budgets [45]. Generating new ideas should include all stakeholders to enhance creativity [46] and leadership plays a vital role [47]. Moreover, in divergent thinking, a creative process helps in discovering novelty [12]. Therefore, creativeness is important for sustainability and change management.

The second explorative BPM skill is “*feeling opportunistic”* because it turned out that experience and knowledge play a significant role in minimizing uncertainty and forming strong opportunistic beliefs [48]. In uncertain conditions, opportunity-related feelings or beliefs seem to help in executing actions in organizations more smoothly. Professional experience is also important for building strong opportunity-oriented feelings [48]. In other words, feeling opportunistic appears to develop a self-confidence for tackling uncertainties.

Thirdly, “*feeling flexible”* is opposed to the efficiency focus of the traditional BPM approach. Explorative BPM mainly emphasizes flexibility in the pre- and post-implementation phase when introducing new business processes or work patterns [49]. Studies have demonstrated that flexible employees can modify processes or tasks on the implementation level, and are able to adapt to those [6]. Therefore, flexible BPM skills can be influenced by changes in the work structures as well [50].

The last explorative BPM skill of “*feeling adaptable to change”* refers to the employees’ capability of working in unusual circumstances or with limited managerial control. For instance, during the COVID-19 teleworking period, employees had to take immediate decisions themselves when their decision makers were not available online. Feeling adaptable to change thus facilitates handling difficult situations. As [51] argued, supply chains can be reconfigured in sudden situations caused by catastrophic events when increasing adaptability. Nonetheless, under rapidly changing circumstances, the employees’ intention to use those skills can increase if employees think that their skills match with the new work patterns.

To explore the impact of actual explorative BPM skills (Section 3.2) on “perceived usefulness of explorative work changes”, we developed a hypothesis with four skills:

H3: Feeling creative (H3a), opportunistic (H3b), flexible (H3c) and adaptable to change (H3d) have a positive impact on one’s perceived usefulness of explorative work changes.

Likewise, to evaluate the impact of actual explorative BPM skills (Section 3.2) on “perceived ease-of-use of explorative work changes”, we derived a similar hypothesis:

H4: Feeling creative (H4a), opportunistic (H4b), flexible (H4c) and adaptable to change (H4d) have a positive impact on one’s perceived ease-of-use of explorative work changes.

Moreover, perceived top management involvement needs extra attention during unusual circumstances. It refers to the employees’ perception of support and motivation from their board or supervisors when they are performing specific tasks, processes or projects [52]. Moreover, top management support is also important in paradigm shifts towards ambidextrous BPM [53]. Regarding the effect of the third independent construct of “perceived top management involvement” on the perceived usefulness of explorative work changes and the perceived ease-of-use of explorative work changes, the following hypotheses have been established:

H5: The perceived top management involvement has a positive impact on one’s perceived usefulness of explorative work changes.
H6: The perceived top management involvement has a positive impact on one’s perceived ease-of-use of explorative work changes.

### 3.3 Extending TAM into an Explorative BPM Acceptance Model (EBAM)

In our work, we mainly used the TAM constructs, as the other social concepts were less important when a technology change is mandatory during lockdowns or post-lockdowns scenarios.

We translated PU into “perceived usefulness of explorative work changes” for referring to explorative work changes that can improve employees’ job performance. For example, working from home can enhance work efficiency by an increased usage of communication tools. On the other hand, PEOU is translated as “perceived ease-of-use of explorative work changes” to link perceptions about easiness to new work changes (e.g., a more flexible and adaptable work environment).

TAM incorporates the impact of PU, PEOU, and external constructs (such as “system characteristics”, “development process”, and “training”) on “intension to use”, as mediators. We verify the impact of three external variables on employees’ acceptance attitudes, namely: (1) actual explorative work features (Section 3.1), (2) actual explorative BPM skills (Section 3.2), and (3) perceived top management involvement (Section 3.2). We deliberately opted for not using a variant of TAM’s “intention to use” because the explorative work changes during the COVID-19 lockdowns were mandatory and rather experimental than centrally imposed. Nonetheless, its behavioral impact is indirectly measured and the employees’ acceptance attitudes serve as the dependent constructs in this study. Table 2 translates the incorporated TAM constructs into our explorative BPM acceptance model (EBAM).

**Table 2:** Linking TAM to EBAM.

Original TAM elements	Proposed EBAM elements
User Acceptance of Information Technology	User acceptance of Explorative Business Process management
External Variables	Actual explorative work features Actual BPM explorative skills Perceived Top Management Involvement
Perceived usefulness	Perceived usefulness of explorative work changes
Perceived ease-of-use	Perceived ease-of-use of explorative work changes
Attitude towards using (A)	Attitudes towards using explorative work adaptations and explorative skills in the future
Behavioral Intention to use	[Not studied, assumed mandatory]
Actual use	[Post-lockdowns]

The COVID-19 lockdowns forced employees to work from home and make more use of digital technologies. More specifically, flexible work routines, working from home and using online meeting tools have become part of daily work. We will scrutinize employees’ attitudes towards explorative work changes to predict the extent to which those work practices are likely to change after the COVID-19 lockdowns in order to become common practice. Since explorative BPM skills (Section 3.2) could be applied more often during the COVID-19 lockdowns, many employees have become able to experience them so that the development of explorative BPM skills might be continued in the post-lockdown period.

Hypothesis development continued to investigate the effect of one’s perceived ease-of-use of explorative work changes on one’s perceived usefulness of explorative work changes, and this based on a similar pattern of TAM [5]. The resulting hypothesis is as follows:

H7: The perceived ease-of-use of explorative work changes has a positive impact on one’s perceived usefulness of explorative work changes.

To determine the importance of one’s perceived usefulness of explorative work changes on the dependent endogenous constructs, we considered dedicated hypotheses as well:

H8: The perceived usefulness of explorative work changes has a positive impact on one’s attitude towards future explorative work (i.e., after the COVID-19 lockdowns).
H9: The perceived usefulness of explorative work changes has a positive impact on one’s attitude towards future explorative BPM skill development.

Finally, to establish the relationship between one’s perceived ease-of-use of explorative work changes and the same dependent constructs, we designed respective hypotheses. Thus:

H10: The perceived ease-of-use of explorative work changes has a positive impact on one’s attitude towards future explorative work (i.e., after the COVID-19 lockdowns).
H11: The perceived ease-of-use of explorative work changes has a positive impact on one’s attitude towards future explorative BPM skill development.

On the right, Fig 1 shows the three independent variables of “actual explorative work features” (Section 3.1), “actual explorative BPM skills” and “perceived top management involvement” (Section 3.2). The TAM-related features of “perceived usefulness of explorative work changes” and “perceived ease-of-use of explorative work changes” have been positioned in the middle of Fig 1 to show a mediating role for increasing the effect of our exogenous latent constructs on “attitude towards future explorative work (i.e., after the COVID-19 lockdowns)” and “attitude towards future explorative BPM skill development”. The research model (Fig 1) allows us to statistically test the hypotheses that translate BPM ambidexterity theory (i.e., particularly the explorative BPM skills) and IT acceptance theory (i.e., the original TAM constructs) to the context of COVID-19 lockdown routines. Following the approach of [54, 55], we do not measure the direct impact of exogenous constructs on endogenous variables (i.e., at the right of Fig 1) because we assumed that both middle constructs are mediating. Notwithstanding, we will discuss the indirect effect after interpreting the results whether a mediating role exists for some constructs.

## 4 Research methodology

We used a quantitative research design for statistically testing Fig 1.

### 4.1 Research design

This study was conducted to investigate the expected changing behavior of employees during and after the COVID-19 lockdowns. It covered cross-sectional data with all responses being collected in a single time span. We followed a Structural Equation Modeling approach (SEM) with partial least square (PLS) regression [56] because our goal was extending the recognized TAM model in the context of explorative business skills and explorative work adaptations. We emphasized latent constructs scores instead of overall goodness-of-fit criteria since our reflective research model contained multiple constructs with underlying indicators (Section 3). Hence, PLS-SEM was a suitable approach to quantify the results [56]. The data analysis comprised descriptive statistics, a measurement model assessment and a structural model assessment.

### 4.2 Ethical statement

We collected data from non-teaching employees thus no children involved, and their personal data were collected but anonymously analyzed and reported. Such personal data were also work-related (thus no medical data, and not involving experiments or human interventions). An ethical committee approval was granted by the ethics committee of the authors’ faculty, with reference no. FEB 2021-G. Informed consent was collected verbally at the start of the survey, including privacy-related information at the first page of the survey.

### 4.3 Survey instrument, sampling and population

A survey instrument was designed with a mixture of existing and self-developed questions, available in Appendix A (Table A1) in S1 File. First, preliminary questions were related to descriptive information such as the respondent’s university name and country, as well as open-ended questions related to the respondent’s use of new technologies in daily routines. The next part covered 61 questions on a 5-point Likert scale (i.e., 1 = strongly disagree; 5 = strongly agree). The questions formulations were partially adopted from related studies (Section 2). For instance, the latent construct of “feeling adaptive” was adapted from [51]. An overview of all related references is provided in Appendix A (Table A2) in S1 File.

The online survey was launched through the Qualtrics platform, which was shared with the selected population through official email addresses. Our population consisted of all administrative employees linked to two universities (i.e., one in Western Europe and one in Asia). This choice was motivated for including two different cultures operating in a similar sector, allowing for a better comparison and for advocating international generalizability. Having similar job descriptions in different cultures also helps to justify that the developed constructs were studied properly in a comparable manner and being generic in nature. For instance, in the work of [57] on the acceptance of multimodal sharing systems in transportation processes, data was also collected from university employees in the context of TAM. Although working in a similar business context, our respondents from universities dealt with various work domains that typify traditional organizations, such as human resources, finance, information technology, data management, knowledge management, etc. Thus, since these work domains are present in almost all organizations and sectors worldwide, we used university employees as a proxy for workers in general. Future research can further validate our work in other sectors such as manufacturing firms. Moreover, since we specifically targeted attitudes towards future explorative work and towards future explorative BPM skills, educational institutions are an appropriate choice as their employees are typically also heavily involved in training, scheduling, skill development and similar tasks.

We used non-probability convenience sampling along with some purposive sampling [57] as only the non-teaching office staff members were selected to scrutinize their work changes and explorative skills. In total, we received 371 responses of which 99 responses were incomplete and 272 complete responses were used in the data analysis phase (i.e., 148 from Western Europe and 124 from Asia). The average time per questionnaire was 20 minutes.

### 4.4 Descriptive analysis and ANOVA-based tests

We first performed a descriptive analysis for job position, gender, age, regional distribution, and the use of IT tools.

While our sample contained a good representation of different age categories (Table 3), Table 4 shows that substantially more women participated in our survey. To verify whether our resulting variables significantly differed for contextual factors, we first conducted ANOVA-based tests with respect to our outcome variables. The assumptions for normality and homogeneity of variance were considered, leading to non-parametric ANOVA-based tests.

**Table 3:** Age frequency (N = 272).

Age	Frequency	Percentage
20–30	74	27.2
31–40	79	29
41–50	71	26.1
51–60	38	14
61 or Above	10	3.7
Total	272	100.00

**Table 4:** Gender ratio (N = 272).

Gender	Frequency	Percentage
Male	97	35.7
Female	174	64
Other	1	0.40
Total	272	100

#### 4.4.1 Assumptions of normality

To test the distribution of our two outcome variables, we applied the Kolmogorov-Smirnov and Shapiro-Wilk tests of normality [58]. Both assume that significance values of less than 0.05 lead to violation of normality. For our two dependent variables (“attitude towards future explorative work” and “attitude towards future explorative BPM skill development”), we tested their normal distribution among all location, gender and age sub groups.

Both tests with location-based sub groups resulted in a *P*-value of 0.001, thus assuming non-normality and a first indication for non-parametric ANOVA-based tests [59]. Similarly, the assumption of normality was violated for the gender sub groups, with significant values of 0.000 for “attitude towards future explorative work” for male and female, and with significant values of 0.009 for male and 0.004 for female regarding “attitude towards future explorative BPM skill development”. All age sub-groups for the first dependent variable were also non-normal, whereas two age sub groups were normally distributed for “Attitude towards skills” and three age sub groups had a non-normal distribution.

#### 4.4.2 Assumptions for homogeneity of variance

The assumptions for homogeneity of variance were studied as another pre-requisite for parametric ANOVA-based tests. Again, we conducted a stepwise analysis of all contextual variables with each dependent variable by using the Levene’s tests for homogeneity of variances [60]. For all location-based sub groups, the results reflected significant values being less than the threshold of 0.05 (i.e., 0.018 and 0.014). Hence, the assumption for homogeneity of variance between subgroups of location was violated.

For the gender sub groups, we obtained different results, assuming equal variances for “Attitude towards work adaptations” (i.e., value of 0.321) and unequal variances for “Attitude towards skills” (i.e., value of 0.04). All age sub group had equal variances, with values of 0.830 and 0.520 respectively. Overall, because homogeneity of variance was only partial, a non-parametric analysis turned out to be more suitable.

#### 4.4.3 Non-parametric analysis

Given the violations of normality and homogeneity in our data, we opted to conduct non-parametric tests according to the type and grouping of variables. The Mann-Whitney U Test [61] was conducted for location (i.e., having two regional groups) while the Kruskal-Wallis Test [62] was applied for gender and age.

The Mann-Whitney U Test for location showed a difference in regional distributions for both dependent variables (i.e., with asymptotic significance value of 0.002 and 0.000, being less than 0.05). On the other hand, the Kruskal-Wallis tests for gender and age confirmed that no statistically significant differences were expected for gender and age. For gender, we obtained significant values of 0.073 and 0.173, respectively. For age, these values were 0.613 and 0.244. To investigate the casual effects of our latent constructs (Fig 1), we continued with PLS-SEM.

### 4.5 Measurement model assessment

The measurement model was assessed through the PLS algorithm in the *SmartPLS3* tool [63]. Internal consistency reliability was ensured through the metrics of Cronbach’s alpha and Composite Reliability (CR), each with a threshold of higher than 0.70 [64]. Next, indicator reliability was established through indicator loadings, which value should also be higher than 0.70 [56]. Loading values above 0.70 determined convergent validity [65]. Additionally, convergent validity was confirmed through Average Variance Extracted (AVE) values, which should be higher than 0.50 [66]. Discriminant validity was assessed with two methods. The first method was the Fornell-Larcker criterion (i.e., the square root of AVE per latent variable should be higher than its highest correlation with other constructs) [67]. Secondly, the heterotrait-monotrait (HTMT) ratio should be less than 0.9 [67]. The measurement model assessment demonstrated the reliability and validity of our reflective measurement instrument [68].

### 4.6 Structural model assessment

The structural model was assessed using collinearity, the coefficient of determination (*R*^*2*^), bias-corrected and accelerated (BCa) bootstrapping and blindfolding-based cross-validated redundancy measures [69]. Collinearity was observed to ensure that there was no multicollinearity in the data. The Variance Inflation Factor (VIF) value should be less than 5, and with a value of less than 3 considered as ideal (i.e., thus free of collinearity problems) [56]. The coefficient of determination (*R*^*2*^) was calculated to predict our model’s explanatory power of endogenous constructs. *R*^*2*^ typically ranges from 0 to 1, and its values of 0.25 (25%), 0.50 (50%) and 0.75(75%) represent weak, moderate and substantial power, respectively [70]. Further on, the predictive relevance of endogenous constructs was examined through a blindfolding test to obtain a cross-validated redundancy measure (*Q*^*2*^), for which a value larger than zero implies that the exogenous constructs have predictive relevance for endogenous variable in a path model [71].

According to [72], bootstrapping is the best procedure for a mediation analysis because the traditional approach of [73] can have limitations in interaction term calculations [63]. Since many scholars have criticized that a direct effect is not necessarily significant in a mediation analysis [55], only bootstrapping the indirect effect appeared to be a suitable method [54]. Hypothesis testing and the assessment of path coefficient significance were measured through the bias-corrected and accelerated (BCa) bootstrapping procedure [69], which involved random sampling repetitions to obtain standard errors for hypotheses [56]. We ran bootstrapping with a pre-specified 5,000 minimum number of bootstraps samples, and observed critical t-values for two-tailed test with a minimum threshold of 1.65 and a significance level at 0.1 [74], which is a common practice in social sciences.

## 5 Results

We first present a text analysis based on the open-ended questions in our survey (Appendix-A, Table A2) in S1 File, before presenting the results of PLS-SEM.

### 5.1 Descriptive text analysis

As we asked respondents about their experience of using communication tools before and during the COVID-19 lockdowns, we observed two comparative trends while working from home. Table 5 shows that mainly Skype, MS teams and ZOOM became more popular than traditional tools (i.e., that employees typically used in the past) and also some new tools were introduced for business purposes (e.g., WhatsApp).

**Table 5:** Communication tools before and during lockdowns (N = 272).

Tools	Tools used before the lockdowns		Tools used during the lockdowns		Trend
	Users	Percentage	Users	Percentage	
Mail	248	91.18	104	38.23	Decreased
telephone	215	79.04	98	36.03	Decreased
Intranet	110	40.44	47	17.28	Decreased
Shared drive	67	24.63	29	10.66	Decreased
Dropbox	37	13.60	17	6.25	Decreased
Skype	61	22.43	74	27.20	Increased
MS teams	77	28.30	204	75	Increased
ZOOM	49	18.01	145	53.31	Increased
Others (e.g., Slack, Starleaf, Teamviewer, Trello, Bongo, Lifesize, and WhatsApp)	25	9.19	39	14.34	Increased

Further on, we asked the respondents to rate the degree of changes in their work routines due to the COVID-19 lockdowns (0 = no work changes; 10 = full of work changes). The mean of 6.48 and standard deviation of 2.19 confirmed the employees’ recognition of change. Likewise, we asked their satisfaction with the work changes triggered by the COVID-19 lockdowns based on a 10-point Likert scale (0 = fully dissatisfied; 10 = fully satisfied). The mean of 6.27 with a 2.15 standard deviation, however, pointed to only a slightly above-average feeling of satisfaction among employees, and thus demonstrating the need for offering organizations some advice about how to increase employees’ acceptance. To further dig into the reasons for these ratings, we performed a content analysis of an open-ended question regarding the traits characterizing these work adaptions. We received a total of 196 valid text codes, which we mapped against the independent constructs related to work changes and explorative BPM skills (Table 6).

**Table 6:** Word counts for characteristics of work adaptations.

Independent constructs	Perceived characteristics of work adaptations	Reference count
**Perceived feature of work modalities**	Communication is easy	[13]
	Work-life balance obtained	[6]
	(Lack of) social contact with colleagues	[6]
	Concentration increased	[4]
	More online courses	[3]
	Enhanced teleworking	[3]
	Everything has been digitalized (e.g., SAP)	[3]
**Perceived feature of work distractions**	Too engaged/involved (so taking a step back is actually good)	[7]
	Feeling Stressed	[6]
	Working with children in the house is very challenging	[5]
	Mood swings	[4]
	Missing interaction with colleagues	[3]
	Need for personal contact	[2]
	Anxiety disorder	[2]
	Less discussions	[1]
	Need for a clean workspace	[1]
	Love challenges	[1]
	Noise in home	[1]
	Lack of quality of communication due to IT-problems	[1]
	No adequate material at home to work with	[1]
	More effort to do normal task	[1]
**Perceived feature of work implications**	Time management	[11]
	Environment (garden, animals) and supportive working environment	[11]
	Efficiency	[9]
	Wish to organize work in a structured way	[5]
	No traffic jams	[5]
	Less stress	[5]
	Self-discipline	[4]
	More sleep	[2]
	Work documents perfectly accessible from home	[1]
	Attention for wellbeing of co-workers	[1]
	Excess programming	[1]
**Feeling creative,**	Creativity gain	[2]
	Openness trait	[1]
**Feeling opportunistic**	Self-confidence and diagnosis	[6]
	Healthier	[2]
	Appreciating the opportunities of having meetings without the travel	[1]
	Fulfillment in getting the job done	[1]
**Feeling flexible**	More independence	[16]
	Flexible feeling	[13]
	Easy to work from home	[5]
	Freedom (no timing, no 9 to 5 job anymore)	[4]
	Comfortable clothes	[1]
**Feeling adaptive to change**	Sociable	[8]
	More control on what you do when	[3]
	Job security	[3]
	Adaptable feel	[1]

We continue with reporting on the PLS-SEM outcomes that computed partial regressions relationships through ordinary least square regressions [66].

### 5.2 Measurement model assessment

Since we developed a measurement instrument with some adapted items, the validity and reliability was checked first. The results of outer loadings, Cronbach’s alpha, CR [75] and AVE are presented in Table 7. Items with less loadings were deleted.

**Table 7:** Measurement model assessment (loadings, Cronbach’s alpha, CR, AVE).

Latent Constructs	Reflective indicator loadings	Cronbach’s Alpha	Composite Reliability	Average Variance Extracted (AVE)
Perceived feature of work modalities		0.826	0.884	0.656
WCM1	0.845			
WCM2	0.825			
WCM3	0.775			
WCM4	0.792			
Perceived feature of work distractions		0.740	0.850	0.654
WD1	0.791			
WD2	0.831			
WD3	0.803			
Perceived feature of work implications		0.718	0.840	0.637
WI1	0.770			
WI2	deleted			
WI3	0.839			
WI4	0.784			
Feeling creative		0.706	0.814	0.525
CR1	0.787			
CR2	0.759			
CR3	0.706			
CR4	0.635			
Feeling opportunistic		0.746	0.854	0.660
OO1	0.788			
OO2	deleted			
OO3	deleted			
OO4	0.837			
OO5	0.812			
OO6	deleted			
Feeling flexible		0.739	0.835	0.560
FX1	0.742			
FX2	0.810			
FX3	0.699			
FX4	0.738			
FX5	deleted			
Feeling adaptable to change		0.767	0.850	0.587
AD1	deleted			
AD2	deleted			
AD3	0.797			
AD4	0.735			
AD5	0.782			
AD6	0.749			
Perceived top management involvement		0.820	0.880	0.648
TM1	0.737			
TM2	0.795			
TM3	0.857			
TM 4	0.826			
Perceived usefulness of explorative work changes		0.827	0.885	0.658
PU1	0.783			
PU2	0.789			
PU3	0.864			
PU4	0.806			
Perceived ease-of-use of explorative work changes		0.754	0.857	0.668
PEOU1	0.756			
PEOU2	0.822			
PEOU3	deleted			
PEOU4	0.870			
PEOU5	deleted			
Attitude towards future explorative work (i.e., after the COVID-19 lockdown)		0.738	0.850	0.654
ATW1	0.848			
ATW2	deleted			
ATW3	0.851			
ATW4	0.721			
ATW5	deleted			
ATW6	deleted			
ATW7	deleted			
ATW8	deleted			
ATW9	deleted			
Attitude towards future explorative BPM skill development		0.818	0.879	0.645
ATS1	deleted			
ATS2	0.836			
ATS3	0.819			
ATS4	0.766			
ATS5	0.791			
ATS6	deleted			
ATS7	deleted			

**Internal consistency reliability** was measured through Cronbach’s alpha (CA) [76], and composite reliability (CR) [77]. The CA values in Table 7 were between 0.70 and 0.90, and thus acceptable [66]. Similarly, all CR values were above 0.8.

**Convergent validity** was ensured through the reflective indicator loadings and AVE values [67]. According to [67], reflective indicator loadings above 0.70 are acceptable, whereas indicator (items) with loadings between 0.40 and 0.70 may be deleted if this deletion will increase composite reliability. By following this guideline, we deleted all items from the respective scale with loading less than 0.70, whereas only CR4 (i.e., question 4 for feeling creative) was retained because it was decreasing the CR value while still being between the 0.40 and 0.70 threshold of keeping, if required. Still, minimum three indicators were used to reflect each construct in the research model. In addition, the AVE value of more the 0.50 was acceptable [56]. In our sample, all AVE values for each construct were greater than the rule of thumb, and thus predicting convergent validity of scale.

**Discriminant validity** was established through the Fornell-Larcker criterion, for which we got similar results as highlighted in Appendix B (Table B1) in S1 File. Appendix B (Table B2) in S1 File also illustrated the HTMT ratio with acceptable value of lower than 0.90 [66], supporting the existence of discriminant validity.

After ensuring internal consistency reliability, convergent validity and discriminant validly, we compiled the results of our structural model assessment.

### 5.3 Structural model assessment

Our structural model was accessed through the standard criteria of BCa bootstrapping, coefficient of determination (R^2^) and blindfolding-based cross-validated redundancy measures [56, 66, 67].

We examined **collinearity** issues before further assessing the structural model. Appendix C (Table C1) in S1 File shows that all VIF values were less than 3, demonstrating no collinearities issues in the data.

Table 8 presents the **coefficient of determination (*R***^***2***^**)** for describing the explanatory power of our model [78]. The construct related to one’s attitude towards future explorative BPM skill development was a dependent construct with an *R*^*2*^ value of 0.321, indicating a good predictive power of 32%. For our second dependent variable (i.e., one’s attitude towards future explorative work), we reached an *R*^*2*^ value of 0.259 (25%), which influence remained relatively weak but still acceptable according to [77]. In addition, the mediating constructs acted as dependent constructs or endogenous construct (i.e., perceived ease-of-use of explorative work changes and perceived usefulness of explorative work changes) and had a 35% and 39% explanatory power, respectively, indicating a moderate level.

**Table 8:** Coefficient of determination (*R*^*2*^).

Endogenous constructs	R Square	R Square Adjusted
Attitude towards future explorative BPM skill development	0.321	0.316
Attitude towards future explorative work (i.e., after the COVID-19 lockdowns)	0.259	0.253
Perceived ease-of-use of explorative work changes_	0.351	0.331
Perceived usefulness of explorative work changes	0.393	0.372

Next, the **blindfolding-based cross-validated redundancy measures** test determined *Q*^*2*^ values (>0) to assess the predictive power of the path model in PLS [79, 80]. The *Q*^*2*^ value for one’s attitude towards future explorative BPM skill development was 0.19, whereas the *Q*^*2*^ value for one’s attitude towards future explorative work was 0.15. The other endogenous constructs for perceived ease-of-use of explorative work changes and perceived usefulness of explorative work changes had *Q*^*2*^ value of 0.20 and 0.24, respectively. More results of the blindfolding test is provided in Appendix C (Table C2) in S1 File. All values thus indicated predictive relevance with values larger than zero [81].

Lastly, since PLS-SEM is a non-parametric procedure, the significance level and t-statistics were determined by bias-corrected and accelerated (BCa) bootstrapping in order to direct our decisions about supported and non-supported hypotheses. In social sciences, the minimum level of significance is less than to 0.10 for accepting a hypothesis in case research has an explorative notion. The results of bootstrapping and hypothesis testing is given in Table 9.

**Table 9:** Bias-corrected and accelerated (BCa) bootstrapping results.

Hypotheses	Original Sample (O)	Sample Mean (M)	Standard Deviation (STDEV)	T Statistics	P Values	Decision
H1a	0.404	0.405	0.058	6.941	0.000	Supported***
H1b	-0.028	-0.041	0.246	0.114	0.909	Not supported
H1c	0.125	0.139	0.238	0.527	0.598	Not supported
H2a	0.213	0.212	0.065	3.283	0.001	Supported***
H2b	0.207	0.188	0.246	0.840	0.401	Not supported
H2c	-0.107	-0.088	0.245	0.435	0.664	Not supported
H3a	-0.111	-0.107	0.060	1.869	0.062	Supported***
H3b	-0.031	-0.031	0.065	0.487	0.626	Not supported
H3c	0.117	0.115	0.067	1.750	0.080	Supported***
H3d	-0.010	-0.009	0.063	0.156	0.876	Not supported
H4a	0.196	0.202	0.066	2.961	0.003	Supported**
H4b	0.032	0.034	0.070	0.449	0.654	Not supported
H4c	0.013	0.016	0.072	0.183	0.855	Not supported
H4d	0.077	0.078	0.084	0.913	0.361	Not supported
H5	-0.077	-0.076	0.064	1.209	0.227	Not supported
H6	0.223	0.221	0.063	3.509	0.000	Supported******
H7	0.330	0.329	0.062	5.369	0.000	Supported***
H8	0.202	0.203	0.071	2.856	0.004	Supported**
H9	0.444	0.447	0.064	6.968	0.000	Supported***
H10	0.381	0.383	0.072	5.274	0.000	Supported***
H11	0.200	0.201	0.066	3.022	0.003	Supported**

* *P*<0.100;

** *P*<0.050;

*** P <0.001.

**Fig 2:**
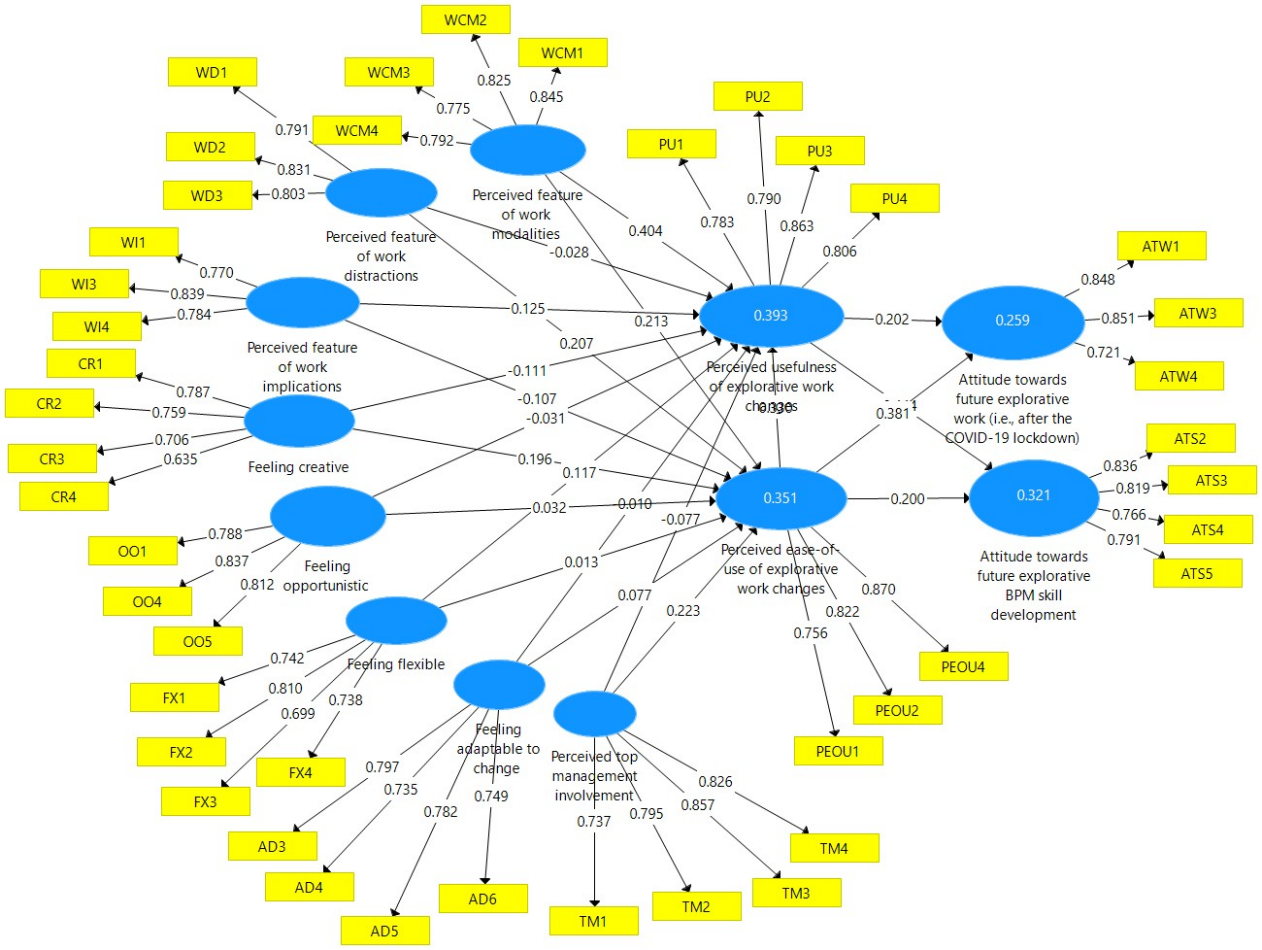
The PLS algorithm output of the explorative BPM acceptance model.

Accordingly, we accepted 11 hypotheses (H1a, H2a, H3a, H3c, H4a, H6, H7, H8, H9, H10, H11), whereas 10 hypotheses were not supported (H1b, H1c, H2b, H2c, H3b, H3d, H4b, H4c, H4d and H5). The final results of the PLS alogrithms (i.e.,in a SmartPLS3 output) are shown as a statistical model in Fig 2.

## 6 Discussion

**Fig 3:**
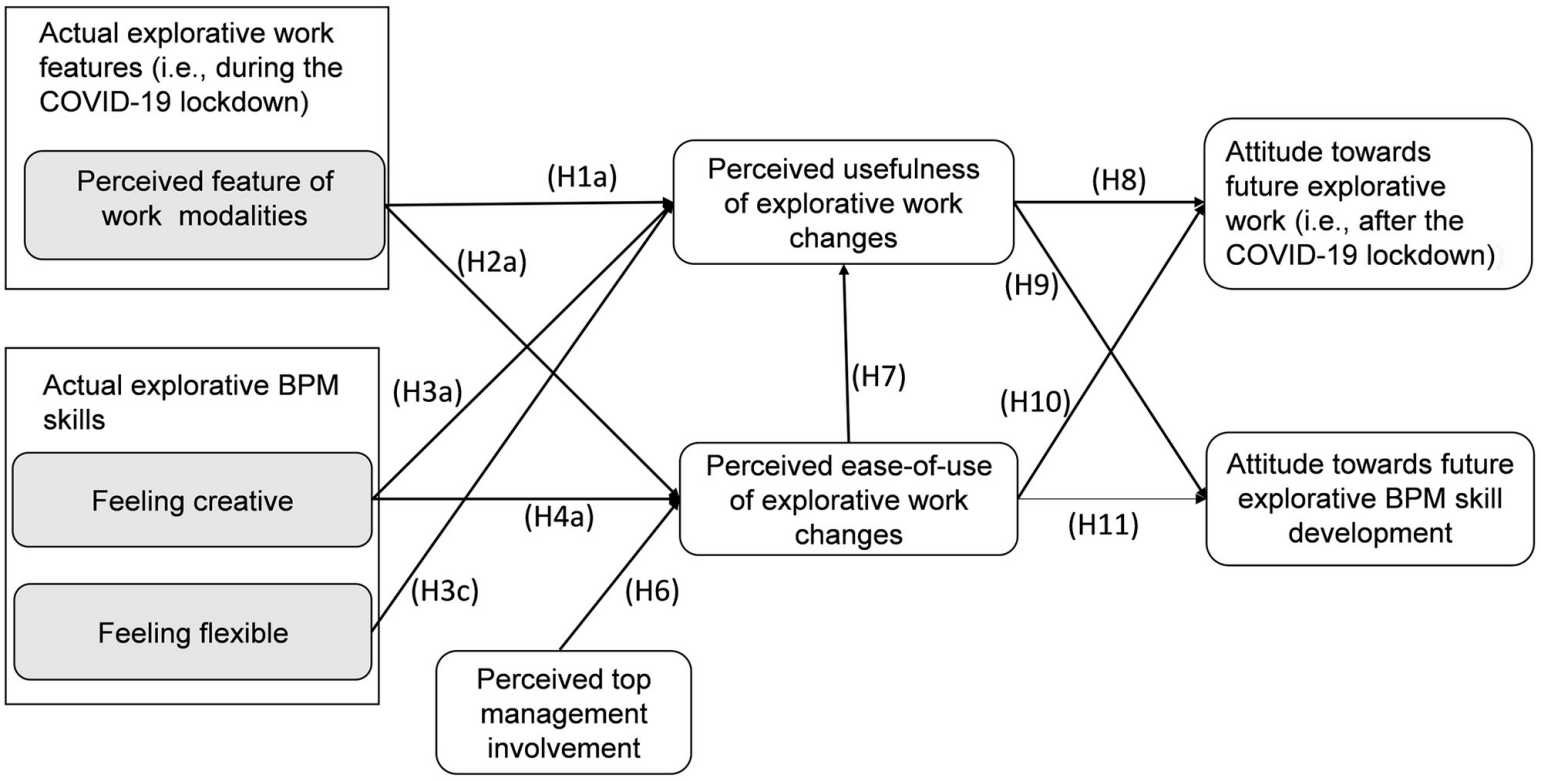
Revised explorative BPM acceptance model (including the COVID-19 lockdowns perspective).

The typical BPM skills used in organizations (e.g., for process modelling, execution, analysis, optimization) are changing according to the new demands in a digital economy. During a series of COVID-19 lockdowns, work routines have been drastically changed into more explorative work features and online communication. For instance, the use of Skype, MS Teams and Zoom has rapidly increased as compared to traditional emails and older commination sources. Similarly, Fig 3 provides evidence (i.e., with accepted hypotheses H1a, H2a, H8, H9, H10 and H11) that the perceived features of work changes (e.g., enhanced use of video/audio commination tools) have positively influenced employees’ attitude towards explorative work.

Our findings reveal that especially the most influencing acceptance factors should be well considered in the context of employees’ attitude development. This work has strengthened a theoretical understanding for linking work changes and a dynamic skill set to acceptance, adoption and behavior. Interestingly, performance is expected to increase, even during lockdowns (when employees are not going to the office but are working from home), under the condition that someone possesses a flexible and creative skill set. On the other hand, top management support is also highly important for explorative mind shaping and accepting fast work changes in dynamic business environments.

### 6.1 Revised explorative BPM acceptance model

Fig 3 visualizes the revised explorative BPM acceptance model. The arrows in Fig 3 summarize which factors help predict employees’ acceptance of explorative work changes and contribute to employees’ interest in further skill development.

Although our prior research model (Fig 1) highlighted distractions and implications under the umbrella of explorative work changes, our PLS-SEM results only supported work modalities. In other words, work modalities (e.g., more ICT adaptation) seem to have a positive influence on the attitude of workers to continue adapting these changes after the lockdown period. As indicated by [24], organizations are reshaping their day-to-day operations and are therefore deviating from routines. On the other hand, work distractions and implications are rather temporary and cannot be considered as affecting in the long run. For instance, teleworking is better if one has family support at home [82]. [82] argued that virtual workspaces also reduce office expenses and increase productivity, whereas distraction is especially a main issue during the long periods of lockdown.

Furthermore, we initially conceptualized four explorative BPM skills (i.e., feeling creative, opportunistic, flexible and adaptive to change) in Fig 1, and then dropped two of them based on statistical evidence (Fig 3). Only feeling creative and feeling flexible appeared to be the most crucial explorative BPM skills that play a role in developing an attitude for practicing work changes and explorative skills. In a recent study [83], linked creativity with ambidexterity and digitization, which confirms that creativity is an explorative feature that can stimulate the acceptance of digital technologies nowadays. In our extended model, both mediating constructs (i.e., PU of explorative work changes and PEOU of explorative work changes) facilitate the relationship between feeling creative and attitudes on the one hand, towards a continued use of explorative changes and skills after a lockdown on the other hand. The arrows for H1a, H2a, H8, H9, H10 and H11 indicate these relationships in Fig 3. Hence, feeling opportunistic and feeling adaptive to change does not seem to significantly affect one’s attitude for future skill development or continued usage.

Nevertheless [84], positioned adaptation as a skill that also includes flexibility and a positive response towards changes and new situations, and they therefore combined the adaptation skill with being flexible and helpful in getting new jobs. Regarding opportunism [85], revealed that opportunism can cause hurdles in a business-to-business value creation because transaction costs increase and the trust factor is suppressed. Hence, opportunistic behavior could violate values, principles, and standards of behavior that have been internalized [86], and which may explain our findings as well.

Furthermore, the need of top management involvement has been confirmed through the PEOU of explorative work changes. Consequently, our revised explorative BPM acceptance model in Fig 3 shows only the statistically significant relationships after a PLS-SEM analysis, which can be an input for future empirical studies as well.

By combining the original TAM [5] constructs with ambidexterity theory [8], we have been able to build and test an explorative BPM acceptance model that comprises actual explorative work features and skills along with employees’ attitudes towards future explorative work and skill development. Another research contribution concerns relating these basic concepts of technology acceptance and ambidexterity with recent advancements in the BPM literature and practices. For instance, our work has extended the explorative BPM skills discussed by [9, 11, 12]. Our work has also shown that an outside-in perspective of BPM is appropriate for environmental scanning to get an optimal benefit of recent communication tools and technologies, even when employees are still reluctant to accept these explorative BPM skills for problem-solving and individual growth. Especially in dynamic business environments (e.g., during crises, natural disasters, pandemics and uncertain competition), these explorative business skills are game changers.

### 6.2 Research and practical implications

This study has contributed to the research domains of BPM and management information systems by exploring factors related to work change modalities in uncertain environments, such as lockdown situations. Other examples that can trigger fast process changes are a political or financial crisis, another pandemic, or emerging technologies. Hence, our study is also linked to risk management and the acceptance of new issues or unexpected changes in how organizations operate. Moreover, the use of TAM concepts in our explorative BPM acceptance model sheds a new light on the notion of ambidexterity in business processes, with perceptions and attitudes being essential for acceptance and adoption. More specifically, we have provided insight into dedicated explorative skills such as feeling creative and being flexible.

We acknowledge that the adoption of ambidextrous BPM needs to be carefully discussed by top management because defining policies and investments in IT are major corporate decisions that play a vital role in creating a balance between exploitative and explorative BPM. Our research advises top managers to examine a suitable skillset for employees. For example, when hiring new employees, having creative and flexible minded people can be preferred. Furthermore, in post-lockdown periods, employees’ attitude are likely to be more favorable towards flexibility and creativity when employees have already positively experienced work change modalities such as online meetings. These new ways of working and thinking should be carefully considered when designing business processes after the COVID-19 pandemic as well. This research calls for BPM practitioners to better cope with adding or eliminating (or rather hibernating) tasks and process activities when the business environment requires fast changes.

### 6.3 Research limitations

Due to the ongoing COVID-19 lockdown circumstances, this study is limited to evaluating employees’ attitudes rather than their actual work change adaptations or their BPM explorative skill trainings in order to provide timely advice and to allow for longitudinal studies (i.e., with this study serving as a reference point). Moreover, we have developed a theoretical model and tested it empirically across two regions to acquire a revised explorative BPM acceptance model. We encourage future works to test this revised model across various business environments and regions. Another limitation concerns our population, as we collected data from universities only (although the non-teaching employees who work in various business functions including human resource management, technology management, communication, finances, and similar to typical organizational departments), in order to further generalize our findings to other sectors. Moreover, although data was collected from two different regions (i.e., Western Europe and Asia), additional continents and business cultures deserve more attention in future research to extend our model.

## 7 Conclusion

Although explorative BPM becomes extremely important when digitally transforming business processes, most organizations are still in an acceptance and adoption phase. We specifically position feelings of creativity and flexibility as crucial for explorative BPM, and showed that these skills can be helpful in accepting the mandatory work change atmosphere caused by several lockdowns during the global COVID-19 pandemic. To uncover additional factors that help predicting employees’ acceptance of explorative BPM, we also considered work-related features of explorative BPM practices and added top management involvement as a relevant factor for boosting the explorative BPM acceptance level. In sum, our revised explorative BPM acceptance model confirms that work change modalities, feeling flexible, feeling creative and top management have most influence on changing employees’ attitude towards future explorative work changes (RQ1) and future skill development (RQ2). Our extensions of the classical technology acceptance model and ambidexterity theory open a gateway for BPM researchers and practitioners to consider more the explorative side of business processes.

## Supporting information

S1 File (DOCX)
