# Supplementary material for: Development and testing of an explorative BPM acceptance model: Insights from the COVID-19 pandemic
Source: PLoS One. 2021 Nov 4;16(11):e0259226. doi: 10.1371/journal.pone.0259226 (PMC8568138; doi:10.1371/journal.pone.0259226)
Supplement: S1 File — (DOCX) [file pone.0259226.s001.docx]

# Supporting information

**S1** [**Appendix A**](https://doi.org/10.6084/m9.figshare.16632409.v1)  **Survey instrument details**

Table A1. Linking constructs and questionnaire items to literature

Table A2. Linking constructs and questionnaire items to Codes and Preliminary questions

(DOCX)

**S2** [**Appendix B**](https://figshare.com/articles/dataset/Appendix_B_docx/16632424) **Results of the measurement model**

[Table B1. Results of the Fornell-Larcker criterion](https://docs.google.com/document/d/1VMSbttSikKqi6zXr5KHDejVVW9wKLa1Pi1w08WDfaKo/edit?usp=sharing)

Table B2. The resulting heterotrait-monotrait (HTMT) ratio

(DOCX)

**S3** [**Appendix C**](https://figshare.com/articles/dataset/Appendix_C_docx/16632430)**. Results of the structural model assessment**

Table C1.The resulting Variance Inflation Factors (VIFs)

Table C2. The resulting blindfolding-based cross-validated redundancy measures.

(DOCX)

**S1** [**Dataset**](https://figshare.com/articles/dataset/Dataset_COVID-BPM_paper_csv/16629163)

(CSV)
